# A Custom-Made Device for Reproducibly Depositing Pre-metered Doses of Nebulized Drugs on Pulmonary Cells *in vitro*

**DOI:** 10.3389/fbioe.2021.643491

**Published:** 2021-04-21

**Authors:** Justus C. Horstmann, Chelsea R. Thorn, Patrick Carius, Florian Graef, Xabier Murgia, Cristiane de Souza Carvalho-Wodarz, Claus-Michael Lehr

**Affiliations:** ^1^Helmholtz Institute for Pharmaceutical Research Saarland (HIPS), Saarbrücken, Germany; ^2^Department of Pharmacy, Saarland University, Saarbrücken, Germany; ^3^Clinical and Health Science, University of South Australia, Adelaide, SA, Australia

**Keywords:** inhalation, aerosol, pulmonary drug delivery, epithelial cells, air–liquid interface

## Abstract

The deposition of pre-metered doses (i.e., defined before and not after exposition) at the air–liquid interface of viable pulmonary epithelial cells remains an important but challenging task for developing aerosol medicines. While some devices allow quantification of the deposited dose after or during the experiment, e.g., gravimetrically, there is still no generally accepted way to deposit small pre-metered doses of aerosolized drugs or pharmaceutical formulations, e.g., nanomedicines. Here, we describe a straightforward custom-made device, allowing connection to commercially available nebulizers with standard cell culture plates. Designed to tightly fit into the approximately 12-mm opening of either a 12-well Transwell^®^ insert or a single 24-well plate, a defined dose of an aerosolized liquid can be directly deposited precisely and reproducibly (4.8% deviation) at the air–liquid interface (ALI) of pulmonary cell cultures. The deposited dose can be controlled by the volume of the nebulized solution, which may vary in a range from 20 to 200 μl. The entire nebulization-deposition maneuver is completed after 30 s and is spatially homogenous. After phosphate-buffered saline (PBS) deposition, the viability and barrier properties transepithelial electrical resistance (TEER) of human bronchial epithelial Calu-3 cells were not negatively affected. Straightforward in manufacture and use, the device enables reproducible deposition of metered doses of aerosolized drugs to study the interactions with pulmonary cell cultures grown at ALI conditions.

## Introduction

The development of drugs against pulmonary diseases requires testing of both safety and efficacy. In this context there recently has been a growing interest in using *in vitro* cell culture models to replace, reduce, and refine animal experiments (Tannenbaum and Bennett, 2015; Ehrmann et al., 2020). Initially, such tests were and still are performed with submerged cell culture models (Pulskamp et al., 2007; Rothen-Rutishauser et al., 2007; Metz et al., 2020). However, as patients inhale drugs as an aerosol, air–liquid interface (ALI) models are more physiologically relevant (Lacroix et al., 2018). It has been shown that testing of aerosolized excipients under ALI conditions is, in many ways, different from testing under liquid-covered conditions (LCCs) (Brandenberger et al., 2010; Paur et al., 2011; Upadhyay and Palmberg, 2018). For instance, drug transport rates across *in vitro* cell culture inserts depend on the donor compartment concentrations and are, therefore, dramatically increased when drugs are applied as dry particles without any additional liquid at the ALI (Bur et al., 2010). Vice versa, adverse effects could be shown at lower doses in ALI conditions compared with LCC, albeit only the nominal—not the cell-delivered dose—would be obtained for submerged culture conditions (Loret et al., 2016). On the contrary, there is also evidence that the culture conditions do not affect the dose-specific efficacy of certain drugs (e.g., bortezomib) in A549 lung epithelial cells (Lenz et al., 2014). Once inhaled *in vivo*, particles tend to land on a layer of mucus or thin lining fluid (e.g., pulmonary surfactant) that is only 1/10 of the particles’ size (Bastacky et al., 1995). Modeling physiological situations when developing models and protocols for meaningful *in vitro* tests is, therefore, pivotal (Bastacky et al., 1995; Hiemstra et al., 2018).

To date, several laboratory methods have already been described to deposit aerosolized drugs on epithelial cells, such as modified impactors or impingers (Cooney et al., 2004; Bur et al., 2009), using electrostatic attraction forces (Jeannet et al., 2016; Frijns et al., 2017) or insufflator devices developed initially for animals (Blank et al., 2006; Bur et al., 2010). Vibrating mesh nebulizers (i.e., Omron NE-U22) have been used to deposit pH-sensitive archeosomes onto macrophages covered with pulmonary surfactant in classic 24-well plates (Altube et al., 2017). While depositing a fine mist onto cell cultures seems trivial, ALI conditions are hardly used, adding complexity to the application. There has also been considerable interest in the pharmaceutical application of dry powders. To study the deposition of metered aerosols from commercially available dry powder inhaler (DPI) devices, systems such as the Pharmaceutical Aerosol Deposition Device on Cell Cultures (PADDOCC) (Hein et al., 2010, 2011) or the Vitrocell^®^ Dry Powder Chamber (Hittinger et al., 2017) have been developed. Other commercially available devices, including the Cultex Devices (Cultex^®^ Technology, 2020), the PreciseInhale^®^, and XposeALI^®^ (Inhalation Sciences, 2020), and the PRIT^®^ System (Fraunhofer P.R.I.T^®^ Systems, 2020), have emerged; and more details are described in recent review articles (Schneider-Daum et al., 2019; Ehrmann et al., 2020). However, the Vitrocell^®^ Cloud systems—originally called Air–liquid Interface Cell Exposure-Cloud (ALICE-Cloud) (Lenz et al., 2014), have become quite popular, as seen in the number of recent publications in both the field of (nano-)particle toxicity (Chortarea et al., 2017; He et al., 2020) and preclinical drug testing (Röhm et al., 2017; D’Angelo et al., 2018). The available standard device consists of a polycarbonate chamber connected to a vibrating mesh nebulizer (Aeroneb^®^ Lab nebulizer unit), generating a cloud of liquid aerosol settling down on multiple Transwell^®^ inserts at the same time. These wells sit in a base module that controls the temperature of the cell medium, and the cell-delivered dose can be determined with a quartz crystal microbalance (Lenz et al., 2009, 2014). Only recently, the Vitrocell^®^ Cloud MAX has been introduced (Vitrocell^®^ Cloud Systems, 2020), which was designed for metered-dose delivery to one Transwell^®^ insert at a time (Cei et al., 2020).

Nevertheless, experimental setups enabling the controlled deposition of predetermined aerosol doses onto one Transwell^®^ insert at a time for exposure of pulmonary epithelial cells under ALI conditions are seldomly available. To close this gap, we here present an easy-to-make and easy-to-use device, consisting of a machined polyoxymethylene (POM) cylinder, which directs a single aerosol dose generated by a vibrating mesh nebulizer (Aeroneb^®^ Lab nebulizer unit) to the bottom of individual wells or inserts of standard multi-well plates. The data presented here demonstrate its suitability to reproducibly deposit pre-metered doses by nebulizing between 20 and 200 μl of an aqueous drug solution and a nanoparticle pharmaceutical drug formulation. Apart from cleaning the device after use, no further maintenance is needed, making it easy to handle under sterile conditions. A proof-of-concept experiment with Transwell^®^ insert-grown Calu-3 cells revealed no signs of cytotoxicity, and the epithelial barrier function as measured by the transepithelial electrical resistance (TEER) was the same as for untreated cells. The system has already been successfully employed earlier by our group for other tasks (Graef et al., 2018) but was so far not further described in detail concerning its construction or application to deposit single doses of drugs on cells.

## Materials and Methods

### Manufacturing of the Chamber and Setup of the System

**FIGURE 1:**
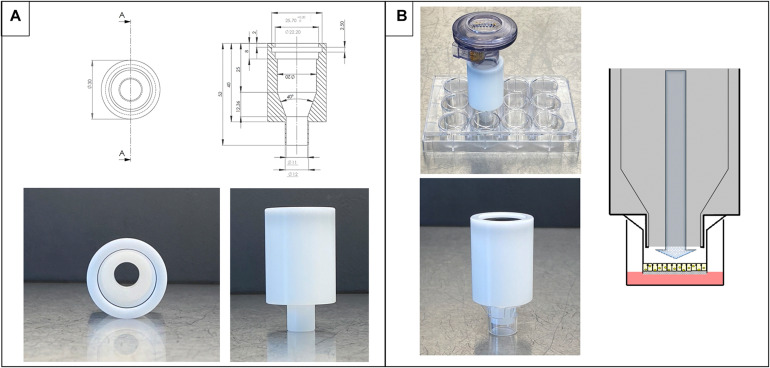
Design and dimensions of the deposition system. **(A)** Technical drawing and images of the device alone. **(B)** Nebulizer connected to deposition device on top of Transwell^®^ inserts ready for nebulization on cells (top image) and details of placement in an insert (bottom). On the right side, schematic view of the process of nebulization in a Transwell^®^ insert.

The deposition device is made of POM and was produced at the workshop of Saarland University (Saarbrücken, Germany). With a standard (computerized numerical control)-milling machine, the cylinder is made from a rod following the dimensions shown in Figure 1A. The cylindrical device has a wider opening to fit on the nebulizer and a smaller opening to fit in a 12-well Transwell^®^ insert, Cat. No. 3460, with a pore size of 0.4 μm (Corning^TM^ Costar^TM^, Lowell, MA, United States, Figure 1B). An Aeroneb^®^ Lab nebulizer unit (standard VMAD, 4.0–6.0 μm droplet diameter) plugged into the deposition device was used and is connected to an Aerogen^®^ USB controller (both Aerogen^®^, Galway, Ireland). The device’s wider opening contains a rim to fit the nebulizer, which stops at the edge of the rim after an 8-mm distance from the entrance (Figure 1A). The rim contains a circular cavity to fit a rubber ring to connect the device in the nebulizing process and prevent aerosol loss. The cylinder itself tapers conically to the opening leading outside to the smaller protruding outlet. This part at the bottom opening is designed precisely to fit the dimensions of a 12-well Transwell^®^ insert (Figure 1B). It does not touch the Transwell^®^ membrane or the well’s walls and leads the aerosol exactly on the apical side of the membrane and not to the basolateral side. Alternatively, the system can also be placed on 24-well plates instead of Transwell^®^ inserts.

### Aerosol Generation and Deposition Protocol

In aerosol deposition studies, sodium fluorescein salt (Sigma-Aldrich) was used at various concentrations (as indicated, 2.5, 25, 100, or 250 μg/ml) diluted in phosphate-buffered saline (PBS; without calcium and magnesium, Sigma-Aldrich, D8537) or loaded into lipid liquid crystalline nanoparticles (LCNPs) as a model pharmaceutical formulation described below. To deposit aerosols, the system is assembled as described. To initialize the nebulization process, 100 μl of PBS was aerosolized three times in the whole system (nebulizer + device). The system is then placed on the respective wells. A volume of 20–200 μl from the desired liquid (or particle suspension) is added to the nebulizer mesh. Once the nebulization process is finished (shown by a small puff of a cloud above the mesh), the nebulizer is kept over the well for another 30 s (or as indicated) to allow the cloud to settle. The nebulizer and the device are separated again, and the remaining solution drops are removed from the downstream side of the mesh membrane by gently wiping with a (sterile) tissue. After the experiments were finished, the nebulizer and device are cleaned with (sterile) deionized water.

To contrast the solution, lipid nanoparticles (LCNPs) loaded with sodium fluorescein (3.5 mg/ml) or tobramycin (5 mg/ml, free base, Sigma-Aldrich) were formed with monoolein (MO; Myverol 18-99K; part number: 5D01253, Kerry Ingredients, and Flavors), as previously described (Thorn et al., 2020). The sodium fluorescein-LCNPs (250 μg/ml) were aerosolized onto Transwell^®^ inserts at volumes of 20–200 μl, to compare with the sodium fluorescein solution. These studies were obtained in another lab as the studies done with free sodium fluorescein to compare the reproducibility of the method. For tobramycin, the aerosolization of a solution was compared with the tobramycin-LCNPs with 200 μl of varying concentrations (0.1, 0.2, 1, and 2 mg/ml) into 24-well plates.

### Drug Deposition Studies in 24-Well Plates

Nebulization with sodium fluorescein (as a surrogate drug) was done as described above. First, parameters affecting drug deposition were changed in order to characterize the system. Two hundred microliters of PBS was filled into 24-well plates, and sodium fluorescein was deposited. Different invested volumes were tested under constant concentration and settling time (20, 50, 100, and 200 μl; 100 μg/ml, 30 s). Different concentrations were tested under constant volume and settling time (2.5, 25, and 250 μg/ml; 20 and 200 μl, 30 s); and different settling times were analyzed under constant volume and concentration (0, 30, and 60 s; 20 μl, 100 μg/ml, and 200 μl with 25 μg/ml). Multiple dosing of the drug was analyzed by nebulizing either 200 μl of 25 μg/ml or 20 μl of 100 μg/ml with 30-s settling time. The deposition was done two and three times in one well.

### Analysis of the Deposited Amounts and Total Recovery of Aerosolized Material

The deposition system was set up as described and placed on Transwell^®^ inserts. Two hundred microliters of PBS was filled on the apical and basolateral sides of the Transwell^®^ inserts to analyze the deposited substance. Basolateral liquid did not touch the bottom side of the Transwell^®^ membrane to prevent free diffusion. Sodium fluorescein or LCNPs in PBS were deposited as described (with 30-s settling time). Afterward, the nebulizer and the device were separated carefully and placed on Petri dishes. Each part was rinsed with 3 ml of PBS in the Petri dishes. One hundred microliters of the apical and basolateral sides and from either the nebulizer or device rinse fluid was withdrawn to analyze deposited mass. The drug deposition efficiency percentage was calculated by the mass of substance in the acceptor well divided by the mass invested in nebulizer times 100.

The fluorescence intensity of sodium fluorescein determined the aerosol-deposited dose measured in 96-well plates at 485-nm excitation and 550-nm emission wavelength with a plate reader (Tecan Trading AG, Infinite M200 Pro). Sodium fluorescein-LCNPs were detected via solubilizing the LCNPs with 0.05% Triton-X and quantifying sodium fluorescein via fluorescent spectroscopy plate reader (Inspire Multimode Plate reader, Perkin Elmer). Similarly, tobramycin was quantified after solubilizing the LCNPs in 0.05% Triton-X in 0.9% sodium chloride and filtration with 4-mm Millex^®^ syringe filters. Liquid chromatography–tandem mass spectrometry (LC-MS) with a Dionex UltiMate 3000 Binary Rapid Separation LC System (Thermo Scientific) coupled with a TSQ Quantum Access Max (QQQ, Thermo Scientific) and a modified ion-pairing method was used for quantification. Trifluoroacetic acid (0.1%), heptafluorobutyric acid (0.1%), and pentafluoropropionic acid (0.1%) were added to eluent A (acetonitrile) and eluent B (water), as a mobile phase. A Zorbax Eclipse xdb C-18 column (5 μm, 50 ^∗^ 4.6 mm, Agilent, Santa Clara, CA, United States) with C18 guard column was used as the analytical column. At a flow of 0.7 ml/min, samples were run with a gradient of eluents A and B, beginning at 20:80 (first minute), changing to 70:30 (1–3.5 min), and restored to 20:80 (3.5–4.5 min). Three microliters of the samples was injected and quantified by positive electrospray ionization (ESI+) and selected reaction monitoring (SRM) of the ion 468.184 → 323.960. A total of nine replicates were analyzed per concentration.

### Viability Testing and Transepithelial Electrical Resistance Measurement After Aerosol Deposition on Calu-3 Cells

The Calu-3 HTB-55 cell line was received from American Type Culture Collection (ATCC^®^) and cultivated in minimum essential medium supplemented with Earle’s salts, L-glutamine, 1% non-essential amino acids (NEAAs), 1 mM of sodium pyruvate, and 10% fetal calf serum (FCS) (all Gibco^TM^, Thermo Fisher Scientific Inc. Waltham, MA, United States). The medium was changed every 2–3 days while using passages between 35 and 55. For experiments, cells were detached using trypsin/EDTA, and 1 × 10^5^ cells were seeded per Transwell^®^ insert. After 3 days, cells were switched to ALI conditions and grown for a total of 11–13 days until being used in the experiments.

Before PBS is aerosolized, the basolateral medium was changed. The controls included wells not exposed to nebulization and inserts with 1% Triton-X 100 (Sigma-Aldrich) in the basolateral medium (control consisting of dead cells). The deposition on cells was done under sterile conditions. Transwell^®^ inserts in a 12-well plate were placed on a heating plate at 37°C. Then, one insert was transferred into a new, empty, 12-well plate with a sterile tweezer, and the aerosolization-deposition maneuver of PBS was performed, as previously described. Permeable supports were placed back to the original well plate filled with 500 μl of medium on the basolateral side. Inserts were incubated at 37°C and 5% CO_2_ for 24 h. Lactate dehydrogenase (LDH) release was assessed with a kit based on color reaction (Roche, Cytotoxicity Detection Kit) from the basolateral medium according to the manufacturer’s advice. The color change was detected with a spectrophotometer (Thermo-Fisher^TM^, Multiskan^TM^ GO) and calculated in % viability of the respective controls. To measure TEER, cells were incubated for another hour at submerged conditions (500/1,500 μl) with the medium. Then, TEER was assessed via electrical Volt-Ohm-meter (EVOM2, World Precision Instruments) with STX2 chopstick electrodes. Values were corrected to the Transwell^®^ insert (1.12 cm^2^) area and the respective value of a blank insert (between 90 and 120 Ω⋅cm^2^). After that, cells were put back to ALI conditions by replacing the medium with 500 μl of fresh medium on the basolateral side and stored in an incubator.

### Nanoparticle Aerosol Deposition Evaluation With the Spatial Distribution

The deposition in the Transwell^®^ inserts was also assessed for spatial distribution using the described method with sodium fluorescein-LCNPs. To ensure no intentional manipulation, directly after sodium fluorescein-LCNP deposition, the membranes were left to equilibrate at room temperature for 1 h. The bottom of the Transwell^®^ insert was then attached to coverslips (#1.5) with Dako Mounting medium (Agilent Technologies). An inverted fluorescent microscope (Olympus IX53) connected to CoolLed pE-300 illuminator system was used with a 2 × objective to visualize the deposition of sodium fluorescein-LCNPs on the membranes, from the bottom side up. Sodium fluorescein was illuminated with the blue-green LED filter and adjusted according to an untreated Transwell^®^ membrane. Three replicates at each volume tested were imaged. ImageJ extracted the fluorescent intensities per pixel across the midline of the membrane’s diameter. For each membrane, four lines were systematically drawn horizontally, vertically, and diagonally in each direction, splitting the membranes into eight parts to obtain an average fluorescent intensity profile. The pixel distances were equated to a numerical distance of the membrane. The pixels’ intensities were converted to a heat map, where the highest intensities were represented by a red color and the lowest intensities by blue. For quantitative analysis, the average fluorescent intensities were correlated to the mean intensity. The intensities were normalized for each volume invested with the highest value in each data equated to an arbitrary value of one and the lowest to zero and plotted against the membrane’s diameter.

### Statistical Analysis

Differences were tested for statistical significance by one-way ANOVA, followed by Tukey’s multiple comparisons test for all solution deposition analyses. The statistical comparison between solution-LCNP formulation and Transwell^®^-well plate inserts were performed by a two-way ANOVA, followed by a Sidak’s multiple comparisons test. *P* < 0.05 were considered statistically significant as described in the respective figure legends. Error bars indicate standard deviation (SD). All statistical tests were performed with GraphPad Prism^®^ 8.

## Results

### Effects of Concentration, Settling Time, and Repeated Deposition

The entire setup consisted of (1) a commercially available nebulizer (e.g., Aeroneb^®^ Lab nebulizer and Aerogen^®^ USB controller) plugged on the (2) custom-made deposition device (as described in the section “Materials and Methods”), which is then (3) placed on either the well of a standard 24-well plate or a 12-well Transwell^®^ insert. The deposition system itself (Figure 1A) is designed not to touch the bottom of the well/Transwell^®^ insert and forms a closed chamber together with the well/Transwell^®^ insert, leaving 5.5-mm distance to the insert or 5.7 mm to the well plate bottom (Figure 1B).

**FIGURE 2:**
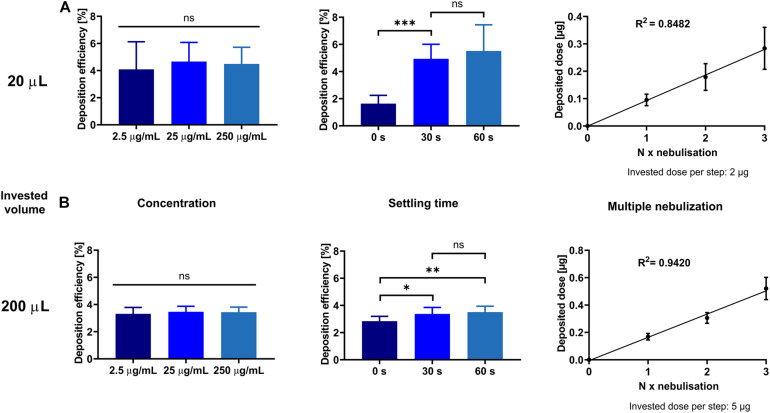
Deposition characteristics at different concentrations, settling time, and multiple nebulization steps. Sodium fluorescein solution is deposited as described in the section “Materials and Methods.” Either 20 μl **(A)** or 200 μl **(B)** was used for nebulization. From left to right, 10-fold increasing concentrations were nebulized, the efficiency of different settling times after the end of nebulization processes was assessed, and respective doses nebulized one or more times into one well at 30-s settling time were analyzed, as indicated. Error bars show standard deviation. One-way ANOVA, Tukey’s multiple comparisons test, ns *p* > 0.12; ^∗^*p* < 0.033; ^∗∗^*p* < 0.002; ^∗∗∗^*p* < 0.001. *N* = 9 of three experiments.

To explore the reproducibility and identify critical factors for aerosol deposition with this device, we investigated the effect of different concentrations, settling times, and multiple depositions (Figure 2). Apart from those factors, the invested volume is the most critical factor, as the generated aerosol deposits directly in a single well. Preliminary trials revealed that invested volumes lower than 20 μl show very high SDs, discouraging the application of smaller volumes (data not shown). This is probably related to the characteristics of the nebulizer’s vibrating mesh, which also propels substance to the apical side of the vibrating mesh. Beginning with 20 μl, the SD of the measured dose for repeated experiments was acceptable in our experiments (22%). At the higher end, 200 μl turned out to be the largest volume to be reproducibly deposited (4.8% SD), since higher volumes lead to condensing drops on the inside wall of the device that dropped out on the well. Notably, these volumes are much smaller than in clinical settings, where volumes of up to 5 ml are used with similar nebulizers (Dolovich and Dhand, 2011; Aerogen^®^, 2020). On account of this, both 20 and 200 μl of invested volumes were further analyzed. A 10-fold change in invested substance concentration did not lead to higher deposition efficiencies at either 20 or 200 μl (*p* ≤ 0.6, Figures 2A,B, respectively).

Regardless of the nebulized volume, longer settling times (time after complete nebulization of invested liquid) had a positive influence on the deposition efficiency, in line with the observation that the generated aerosol cloud is still settling after the end of the nebulization itself. When nebulizing 200 μl, a 30-s settling time was found to be necessary, but further increasing it to 60 s did not significantly improve deposition efficiency (Figures 2A,B). In the case of 20 μl, which takes only about 3 s for nebulization, the benefit of a 30-s waiting time became still more prominent and was therefore adopted as routine for the protocol.

To show that a distinct dose is precisely deposited and can even be enlarged linearly by its increment, multiple repeated depositions were performed for both small and high volumes (Figure 2). After each respective nebulization step, the nebulized dose was added up and reproducibly deposited multiple times to achieve the desired dose. Even so, *R*^2^-values show a more precise deposition with 200 μl of volume than with 20 μl (0.9420 vs. 0.8482).

### The Deposited Mass Linearly Depends on the Invested Volume

**FIGURE 3:**
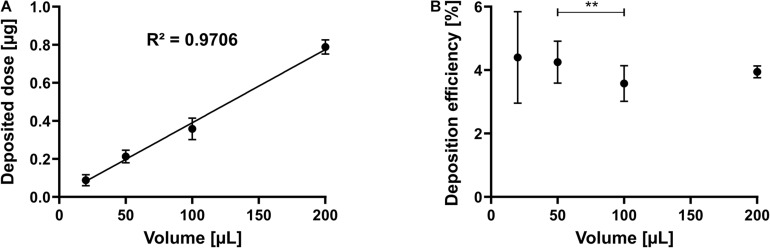
Linearity by dose and efficiency. Sodium fluorescein solution is nebulized as described in the section “Materials and Methods.” **(A)** Deposited dose of a 100 μg/ml solution at different invested volumes. **(B)** Deposition efficiency at different invested volumes. Error bars indicate standard deviation. *N* = 18 of six experiments (for 20 and 200 μl) and *N* = 21 of seven experiments (for 50 and 100 μl). One-way ANOVA, Tukey’s multiple comparisons test; **p = 0.002.

After identifying the range of possible volumes between 20 and 200 μl and the necessary settling time of 30 s, we asked if the volume of the nebulized solution could control the deposited amount of a dissolved compound. Hence, the system was tested with increasing volumes of sodium fluorescein (100 μg/ml) to confirm this hypothesis. Six repetitions were performed and analyzed in triplicate for each volume tested, yielding 18 observations for each data point. As shown in Figure 3A, the deposited dose increased linearly and thus can be controlled by the invested volume (R^2^: 0.9706). Not surprisingly, calculating and plotting the deposition efficiency of the same dataset show that the smaller the invested volume, the higher the SD (1.44 for 20 μl and 0.18 for 200 μl) (Figure 3B). Nevertheless, the system allows to deposit a finite pre-metered dose with reasonable reproducibility, and the invested volume may control this dose.

### Mass Balance Reveals the Distribution of the Deposited Substance in the System

Consistently, about 4% of the nebulized dose was deposited in the well. Therefore, the question arises where the rest of the nebulized substance goes. Besides the mass deposited on the apical side of a Transwell^®^ insert, we also quantified the amounts deposited in the device itself and remaining in the nebulizer after loading it with 20, 100, and 200 μl of a 25 μg/ml sodium fluorescein solution (Table 1). With increasing volume, the relative amount of deposited mass in the device increased from 46 to 63%, as did the relative amount remaining in the nebulizer (from 21 to 27%). The respective amounts of mass deposited on the inserts remained around 4%. As calculated by the sum of the amounts collected in all three compartments (=total recovery), the total recovery was 80% after nebulization with 20 μl but increased to 93 and 94% after nebulization of 100 and 200 μl, respectively. After deposition on the apical side of the Transwell^®^ insert, no substance was found on the basolateral side, confirming that the tapered cylinder structure restricts the deposition to the apical side (data not shown). As described, this is tested in Transwell^®^ inserts with a pore size of 0.4 μm and without contact to basolateral medium to avoid free diffusion.

**TABLE 1:** Total recovery of substance in the system after nebulization and deposition on the apical side of permeable supports.

	20 μl	100 μl	200 μl
Nebulizer	20.5 ± 12.2	34.2 ± 2.83	27.2 ± 3.64
Device	45.8 ± 9.37	55.4 ± 3.32	63.0 ± 8.77
Transwell	5.52 ± 0.84	3.31 ± 0.54	3.43 ± 0.23
Total recovery	79.7 ± 9.02	92.9 ± 2.02	93.5 ± 8.51

*20, 100, and 200 μl of sodium fluorescein solution are nebulized at 25 μg/ml as described in the section “Materials and Methods.” The relative abundance of deposited substance in each part of the system is displayed for each nebulized volume. “Total recovery” is the sum of the relative abundance of each invested volume. Error represents standard deviation. N = 9 of three experiments; 100 μl, N = 6 of three experiments.*

### Analysis of Reproducibility of Deposition Between Free Drug and Particles and Well Plates

**FIGURE 4:**
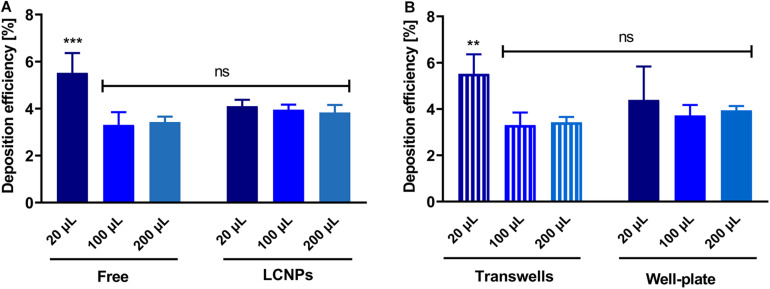
Comparison of deposition efficiency of different volumes, substances, and wells. **(A)** Deposition efficiencies of free sodium fluorescein and LCNPs loaded with sodium fluorescein. Substance deposited on the apical side of permeable supports was analyzed (the section “Materials and Methods”), *N* = 9 of two experiments (for 20 μl of free and LCNP, 100 and 200 μl of LCNP); *N* = 6 of two experiments (for 100 μl of free); *N* = 8 of three experiments (for 200 μl of free). **(B)** Comparison of deposition efficiency on 24-well plates and Transwell^®^ inserts. Sodium fluorescein was nebulized using the device as described. Either the device deposited on Transwell^®^ inserts or 24-well plate inserts. Data show mean and standard deviation. *N* = 9 of three experiments; 100 μl, *N* = 6 of two experiments. Two-way ANOVA, Sidak’s multiple comparisons test; ***p < 0.001; **p < 0.003; ns, no significant difference (*p* > 0.05).

**FIGURE 5:**
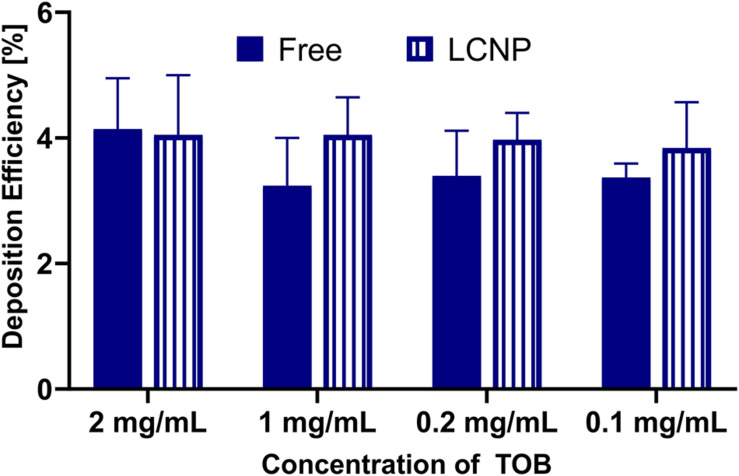
Comparison of deposition efficiency of free and LCNP encapsulated tobramycin (TOB), either as a free solution or in LCNPs after nebulizing 200 μl with Aerogen^®^ Pro nebulizer and nebulization chamber into 24-well plates. Data represented as mean ± standard deviation, *N* = 9 (of three experiments). Two-way ANOVA, Sidak’s multiple comparisons test. No significant difference (*p* > 0.05) was found between the groups.

The deposition of sodium fluorescein as an aerosolized solution or in a pharmaceutical formulation (i.e., LCNPs) was compared to evaluate the robustness of using the device for other applications in a wider pharmaceutical field. Deposition of sodium fluorescein was performed in another lab than the deposition of sodium fluorescein formulation (Lab 1: Helmholtz Institute for Pharmaceutical Research Saarland; Lab 2: University of South Australia). When investing 20, 100, or 200 μl, sodium fluorescein’s deposition efficiency as a free solution or in LCNPs was comparable (Figure 4A). While only the 20 μl of free sodium fluorescein showed a slight, statistically significant increase, all other groups showed a deposition efficiency that was well comparable. The same trend was observed by comparing the deposition of free sodium fluorescein into a 24-well plate and Transwell^®^ inserts, which was essentially the same, except for the 20 μl deposition into Transwell^®^ (Figure 4B). The variation in the accuracy of pipetted micro-volumes increases toward lower volumes, which may further explain the variation observed at 20 μl of invested volume. Compared with a different compound (i.e., tobramycin), the deposition efficiency remained consistent at ∼4% (*p* > 0.05, Figure 5) across 0.1–2 mg/ml invested concentrations at 200 μl of invested volume, in both conditions of a solution and LCNPs. This is the same as sodium fluorescein deposition efficiency, proving the usability of this surrogate substance. Generally, the deposition of different formulations and drugs on different well plates demonstrates the high versatility of using this device.

### Homogeneity of Deposition

**FIGURE 6:**
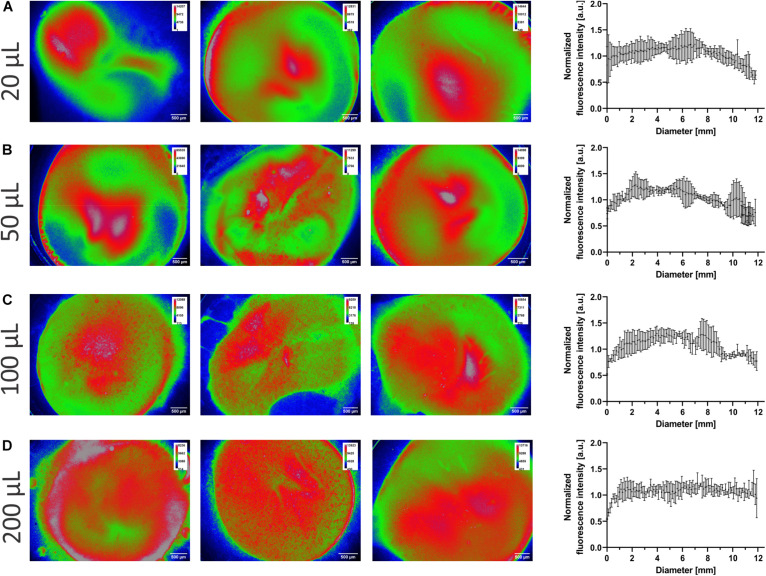
Homogeneity of deposition. Sodium fluorescein-liquid crystalline nanoparticles (LCNPs) were nebulized onto Transwell^®^ insert membranes as described in the section “Materials and Methods.” **(A–D)** Representative fluorescent micrographs of the membranes (from the bottom up), where the highest fluorescence intensities are color-coded as red and the lowest as blue. The fluorescence intensities per pixel were extracted across the center of the membrane. The individual intensities (correlated to the mean intensity) were normalized to arbitrary 1 and plotted against the diameter of the membrane. *N* = 3, data are reported as mean with standard deviation, where every 25th data point is shown for clarity. a.u, Arbitrary units.

Control over the amount of aerosol deposited is essential, so too is the aerosol evenly spread over the surface. The sodium fluorescein-LCNPs were aerosolized onto Transwell^®^ insert membranes (area of 1.12 cm^2^) at 20–200 μl to determine the deposition’s homogeneity. Extraction of the fluorescent intensities of sodium fluorescein across each pixel of the membrane’s diameter provided a quantitative analysis that was normalized for comparison (as indicated). The sub-200-nm particles are evenly spread across the Transwell^®^ membranes, as quantified by the trend in the normalized intensity data in Figures 6A–D. The heat maps of each individual membrane depict the whole spatial deposition and dictate greater heat spots toward the center of the membranes that spread toward the edges. The visual representation suggests an increase in the spread of the particles across the membrane from 20 up to 200 μl, which does not correlate to a difference in spatial homogeneity from the (normalized) quantified data. While the smaller invested volumes have overall lower proportions of red areas, this may reflect the lower dose deposited compared with the higher volumes. There was no statistical difference (*p* = 0.945) between the normalized mean across the diameter, indicating similarities in the homogenous spatial distribution from all four doses. The mean deposition across the diameter was consistent across all volumes invested, normalized to 1.04, 1.02, 1.08, and 1.07 AU for 20, 50, 100, and 200 μl, respectively, indicative of a consistent maximum dose of the compound that was spread homogenously across the membrane. On average, the SD between samples was 7, 12, 10, and 9%, respectively, for 20–200 μl of investment. Even though 10 × more mass is invested, the SD did not severely change and further highlighted the device’s robustness depositing spatially even pre-metered doses.

### Deposition on Epithelial Cells Is Well Tolerated

**FIGURE 7:**
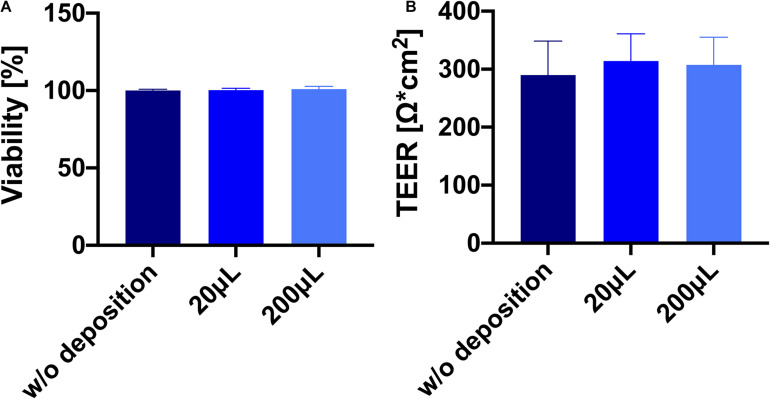
Deposition of phosphate-buffered saline (PBS) on Calu-3 cells grown in Transwell^®^ inserts is well tolerated. **(A)** Viability of Calu-3 cells [Lactate dehydrogenase (LDH), see section “Materials and Methods”] 24 h after deposition of PBS. **(B)** Barrier properties of Calu-3 cells 24 h after deposition. *N* = 9 of three individual experiments. No significant difference was found between the groups (*p* > 0.05).

To demonstrate that the nebulization-deposition maneuver itself with the new device is not noxious to pulmonary epithelial cells, either 20 or 200 μl of PBS was nebulized on the widely used human bronchial epithelial cell line Calu-3, which forms tight monolayers at ALI conditions (Foster et al., 2000; Schneider-Daum et al., 2019). The cells did not show any loss of viability as measured by LDH release (Figure 7A). The TEER as an indicator for the epithelial barrier function remained unchanged as well (Figure 7B).

## Discussion

Here, we describe a new, custom-designed device intended for aerosol deposition into single Transwell^®^ inserts for drug delivery applications of *in vitro* cell culture models. It consists of a tapered cylinder design, which is very compact and connects to commonly used nebulizers (Figure 1). The Aeroneb^®^ Lab nebulizer produces an aerosol cloud into the device that enables a precise and reproducible deposition of a pre-metered dose into the respective well (Figure 1B). The device can be used for single experiments nebulizing one dose on one or more inserts, or the device can be employed to deposit more than one dose on one insert. Due to its low price, many devices can be used without the necessity to clean them during time-critical experiments, as there are examples in the literature comparing many substances instead of using only single agents (Meindl et al., 2015; Röhm et al., 2017; Barosova et al., 2020).

There are two commercially available devices from Vitrocell^®^ Systems that also allow for single insert exposure using an Aeroneb^®^ Lab nebulizer and a cloud-settling principle for dose-controlled aerosol delivery, as comparable with the one presented here. These include 1) the Vitrocell^®^ Cloud MAX and (2) the so-called “Starter Kit.” However, both systems differ significantly from the device described here, as they offer extensive technical features such as an integrated microbalance to determine the post-metered dose and are significantly more costly. The “Starter Kit” design is comparable with the Cloud Systems with a rectangular aerosol-cell exposure system (Lenz et al., 2014). Rather than exposing an entire well plate with several Transwell^®^ inserts at a time, the chamber is smaller (ca. 1 L) to expose a single Transwell^®^ insert (Di Cristo et al., 2020; Vitrocell^®^ Cloud Systems, 2020). The former, the Vitrocell^®^ Cloud MAX, had been introduced a few months ago, and its performance has been described for a prototype version in the literature (Cei et al., 2020). Its exposure chamber has a compact cylindrical design with roughly comparable dimensions to the device reported here (40–60 mm height, diameter ca. 20 mm) tailored toward providing just enough space for one 6-well Transwell^®^ insert (or a smaller-sized insert), with a settling time of ca. 1 min. The bottom part of the cylindrical chamber is not tapered to a diameter of 12 mm (12-well Transwell^®^ insert), and the Transwell^®^ insert has to be put into a base module for exposure. The Vitrocell^®^ Cloud MAX system comes with three or six exposure units arranged in parallel.

Despite some commonalities with existing deposition systems, the crucial advantage of the cost-effective and straightforward custom-made device presented here is the precise ability to control and predetermine the exact deposited dose achieved, as would be done in the clinic (Dolovich and Dhand, 2011). The deposited dose increases linearly with the invested volume (Figure 3A), where micro-sized volumes can be efficiently deposited and do not differ between drugs or pharmaceutical formulations. The system also allows for consecutive dosing to the cells for any invested volume (Figures 2A,B). Thus, the simple design and the low-cost production of the present device allow for reproducible drug deposition as an aerosol *in vitro*.

While a deposition efficiency of about 4% may appear relatively low, it is sufficient for performing meaningful *in vitro* studies, where the amount of compound needed is much smaller than for *in vivo* studies. By increasing the settling time, higher deposition efficiencies can be achieved (Figure 2). Still, we recommend using only 30 s, as the deposition efficiency is not significantly higher (*p* = 0.60). It is more important that the absolute dose is well controlled, as widely observed with our device. In comparable studies with the Vitrocell^®^ Cloud MAX system prototype version, a drug delivery efficiency of 52% was reported, albeit for a six-well Transwell^®^ insert (Cei et al., 2020). By extrapolating these data to smaller inserts/wells, it may be expected that for 12-well Transwell^®^ inserts, the delivery efficiency is about 4.5-fold lower (ca. 12% delivery efficiency), since the surface area of a 12-well Transwell^®^ insert is about 4.5 times less than a six-well Transwell^®^ insert. By using a similar nebulizer and the ALICE/Vitrocell^®^ Cloud system, a deposition efficacy of about 17% was reported (Lenz et al., 2014), but this refers to the simultaneous deposition of an entire six-well plate and needs to be divided by the respective number of wells, which equates to an approximate 3% deposition efficiency per well.

Moreover, Di Cristo et al. (2020) have also recently used the newer Vitrocell^®^ Starter Kit, investing 125 μl of a 1 mg/ml particle suspension. From these data, one can calculate a deposition efficiency per well (1.12 cm^2^) by dividing the deposited amount by the invested amount, yielding an efficiency of 0.64% per well (1.12 cm^2^). This value, which is lower than what we report in the present study, is likely attributable to the larger space that the cloud is nebulized in and the larger surface area for deposition, which is the space around the insert and the walls of the device.

None of the previous studies further investigated the fraction of the lost aerosol during the nebulization process. In this study, it was hypothesized that most of the aerosol lands in the cylinder device. Indeed, with elevating the nebulized dose, more than half of the substance deposits in the cylinder [46% (20 μl) vs. 64% (200 μl)]. This finding explains the already mentioned upper limit of possible nebulized volume (see section Effects of Concentration, Settling Time, and Repeated Deposition). Nebulization of more than 200 μl leads to condensing drops to fall, foiling the intended use at ALI conditions. Regardless, as long as it remains consistent, the deposition of aerosol droplets on the device’s walls is not a clinically relevant problem in practice. By gently wiping the device with (sterile) tissues, repeated nebulization-deposition maneuvers can be done in a row. According to the deposition efficiencies, the total maximum volume deposited onto the wells/inserts was never more than 8 μl, challenging to spread evenly using a pipette without touching the cells.

As could be expected, the deposition of aerosolized saline was well tolerated by commonly used Calu-3 cells, which is in concordance with comparable devices following the same principle, as there are no impaction forces or drying processes (Lenz et al., 2009). The device is usable under sterile experimental circumstances, as it is easily cleanable with ethanolic disinfectant and can be autoclaved with steam. Both LDH release and TEER values show no differences to the control that was not deposited with PBS at either 20 or 200 μl after 24 h (Figure 7). Epithelial cells and the biological absorption barrier formed by their tight junctions must not be harmed following deposition, especially when creating infected or inflamed models and then treated. The spatial distribution snapshot demonstrates an almost-even distribution of the aerosol, as represented in Figure 6, across 20–200 μl of invested dose and further suggests that cell cultures will be exposed to an even dose.

Here, we have visually shown and quantified the fluorescent intensity of nanoparticles deposited onto Transwell^®^ membranes. The precise spatial distribution was observed on a non-wetted membrane that was not tampered with during the nebulization and imaging processes. The particles are homogeneously spread from the quantification of the normalized fluorescent intensity across the membrane’s diameter. This is in agreement with other devices, such as ALICE, which produced a spatially homogenous spread of zinc oxide nanoparticles (Lenz et al., 2009), while in ALI cell culture conditions, the membrane may be wetted from the basolateral compartment and lining fluid of the cells, and this would lead to a greater spread of the aerosol over time. Our snapshot dictates that the aerosol spreads evenly on a dry membrane and does not need to rely on the surface’s wettability. Comparatively, the naturally dried membrane may have resulted in small amounts of crystal formation from the deposited dose resulting in some small artifacts in the micrographs. The consistent ca. 10% SD of the deposited dose across all invested volumes tends to be higher than that of other reports from the Vitrocell^®^ Cloud systems (Ding et al., 2020); however, it may be indicative of the fluorescent microscopy imaging technique as opposed to quartz crystal microbalance quantification. In any case, the device deposits a robust, spatially homogenous dose.

The present paper describes a straightforward device, in both manufacture and use, that enables reproducible deposition (4.8% relative SD) of pre-metered doses of aerosolized drugs on pulmonary *in vitro* cell cultures grown at ALI conditions. With this device, volume-defined amounts of solubilized drugs and pharmaceutical aerosol formulations can be deposited precisely on wells. The distribution of the deposited mass of free drug could be analyzed throughout the whole system. As expected, the deposition, when using this device on cell culture inserts, does not interfere with cell viability and epithelial barrier function. It is easy to clean, cost-efficient, and easily transferable to the bench. It can be customized to connect to any nebulizer and is the only device that could be completely produced using 3D printers, a technology that is employed universally at most universities in the world. Therefore, it can provide a valuable tool for studying the effects of aerosolized drugs and nanoscale delivery systems on *in vitro* pulmonary cell culture models.

## Data Availability Statement

The raw data supporting the conclusions of this article will be made available by the authors, without undue reservation.

## Author Contributions

JH performed the experiments and wrote the manuscript. CT performed the LCNP experiments, including the data analysis of deposition efficiency and spatial deposition of the aerosol and revising the manuscript. PC helped in the setup and design of the deposition chamber and technical questions and in revising the manuscript. XM and FG had the initial idea of producing the cylinder, planned the concept, and developed the device. CC-W advised on experiments and strategy and helped to create the manuscript and to enhance it. C-ML provided help in planning the experiments and supervised, creating, and enhancing the manuscript. All authors contributed to the article and approved the submitted version.

## Conflict of Interest

The authors declare that the research was conducted in the absence of any commercial or financial relationships that could be construed as a potential conflict of interest.
